# Mapping evidence of intervention strategies to improving men’s uptake to HIV testing services in sub-Saharan Africa: A systematic scoping review

**DOI:** 10.1186/s12879-019-4124-y

**Published:** 2019-06-06

**Authors:** Mbuzeleni Hlongwa, Tivani Mashamba-Thompson, Sizwe Makhunga, Khumbulani Hlongwana

**Affiliations:** Discipline of Public Health, School of Nursing and Public Health, University of KwaZulu-Natal, Durban, South Africa

## Abstract

**Background:**

HIV testing serves as a critical gateway for linkage and retention to care services, particularly in sub-Saharan African countries with high burden of HIV infections. However, the current progress towards addressing the first cascade of the 90–90-90 programme is largely contributed by women. This study aimed to map evidence on the intervention strategies to improve HIV uptake among men in sub-Saharan Africa.

**Methods:**

We conducted a scoping review guided by Arksey and O’Malley’s (2005) framework and Levac et al. (2010) recommendation for methodological enhancement for scoping review studies. We searched for eligible articles from electronic databases such as PubMed/MEDLINE; American Doctoral Dissertations via EBSCO host; Union Catalogue of Theses and Dissertations (UCTD); SA ePublications via SABINET Online; World Cat Dissertations; Theses via OCLC; and Google Scholar. We included studies from January 1990 to August 2018. We used the PRISMA extension for scoping reviews (PRISMA-ScR): checklist and explanation. The Mixed Method Appraisal Tool version 2018 was used to determine the methodological quality of the included studies. We further used NVivo version 11 to aid with content thematic analysis.

**Results:**

This study revealed that teaching men about HIV; Community-Based HIV testing; Home-Based HIV testing; Antenatal Care HIV testing; HIV testing incentives and HIV Self-testing are important strategies to improving HIV testing among men in sub-Saharan Africa. The need for improving programmes aimed at giving more information to men about HIV that are specifically tailored for men, especially given their poor uptake of HIV testing services was also found. This study further revealed the need for implementing Universal Test and Treat among HIV positive men found through community-based testing strategies, while suggesting the importance of restructuring home-based HIV testing visits to address the gap posed by mobile populations.

**Conclusion:**

The community HIV testing, as well as, HIV self-testing strategies showed great potential to increase HIV uptake among men in sub-Saharan Africa. However, to address poor linkage to care, ART should be initiated soon after HIV diagnosis is concluded during community testing services. We also recommend more research aimed at addressing the quality of HIV self-testing kits, as well as, improving the monitoring systems of the distributed HIV self-testing kits.

**Electronic supplementary material:**

The online version of this article (10.1186/s12879-019-4124-y) contains supplementary material, which is available to authorized users.

## Background

HIV testing serves as a critical gateway for linkage and retention to care services. This is also key to improving the health and well-being of populations infected with HIV. While it is clear that linking HIV testing with direct linkage and retention to care remain paramount in addressing the 90–90-90 strategy (90% of all people living with HIV should be diagnosed, 90% of people diagnosed with HIV are started on Antiretroviral treatment (ART), and 90% of people started on ART have a suppressed viral load), attracting men remain one of the priorities of this programme [1]. Although many intervention strategies have been implemented in Africa to improve HIV testing and ART initiation [2–8], men have not responded well to these initiatives, compared to their female counterparts [9]^,^ thereby exposing more women to new HIV infections, as well as, reversing the progress made on addressing the HIV epidemic [10]. Studies have shown that attracting men for HIV testing services in the first place has been the main challenge because very fewer men are found and consented to HIV testing services [11–13]. Even the scale-up of HIV testing and treatment cascade has largely benefited women than men, further growing female-male disparity in adult life expectancy [14].

While studies have been conducted to understand barriers to HIV testing and linkage to care (LTC) in Africa [15–18], few collated evidence on intervention strategies for improving HIV testing among men in the sub-Saharan African (SSA) continent using a systematic scoping review [7, 19–23]. This study maps evidence on the intervention strategies implemented to improve HIV case-finding among men in SSA.

To address the challenges facing men in utilising HIV testing services, many intervention strategies have been implemented in Africa, with noticeable progress to improving HIV uptake among men, including home-based HIV testing [2–4], couples testing during Antenatal Care (ANC) visits [24], provider-initiated testing and counselling (PITC) [25], as well as LTC testing [26]. HIV self-testing (HIVST) has also been introduced as an additional strategy to improve men’s HIV uptake in some African countries [27]. However, there remains a gap in systematically appraising these intervention strategies in Africa, especially with specific focus to men, as they are hard to reach [9]. While this is the case, the health outcomes between men and women is rather widening [28, 29] and this suggests that male-centred intervention strategies to engaging men on HIV testing and ART initiations are required. This study aims to systematically map evidence of interventions and strategies aimed at improving men’s uptake to HIV testing services in SSA over a period spanning from 1990 to 2018. Therefore, our research questions was: what are intervention strategies aimed at improving men’s uptake to HIV testing services in SSA? We included all studies found, by our research method, as having been published between the years 1990 to 2018 because studies published prior to 1990 are unlikely to reflect the key aspects and changes pertaining to Human Immunodeficiency Virus (HIV) and Acquired Immunodeficiency Syndrome (AIDS). More studies were conducted after 1990 and many interventions and guidelines were implemented to address the HIV/AIDS epidemic.

## Methodology

### Design

We conducted a scoping review of published peer-reviewed and grey literature (literature non-formally published scholarly or substantive information) studies on the intervention strategies to improving men’s uptake to HIV testing services in SSA. Methods for this study were guided by Arksey and O’Malley’s (2005) scoping review framework [30], and Levac et al. (2010) methodological enhancement for scoping review projects [31]. According to Arksey and O’Malley’s framework, there are five different stages in undertaking a scoping review: (1) identifying the research question; (2) identifying relevant studies; (3) selecting studies; (4) charting the data and (5) collating, summarising and reporting the results. We also followed the PRISMA extension for scoping reviews (PRISMA-ScR): checklist and explanation [32]. The Population, Concept and Context (PCC) framework was also employed in this study to determine the eligibility of research question (Table 1).

**Table 1:** PCC Framework

Criteria	Determinants
Population	Men of all age groups in SSA
Concept	HIV testing among men, intervention strategies on HIV testing, knowledge about HIV
Context	HIV/AIDS

### Eligibility of research question

The Population, Concept and Context (PCC) framework was employed in this study to determine the eligibility of research question (Table 1). The framework indicate that our study’s population was men of all age groups in SSA, while our concept was HIV testing. The interventions employed in the included studies involved education about HIV; testing men for HIV at community level and at homes, encouraging men to test for HIV during ANC visits, offering incentives to attract men to test for HIV and encouraging men to test for HIV when after their sexual partners have been diagnosed with HIV.

### Identifying relevant studies

The article searches were inclusive of databases, such as, PubMed/MEDLINE, American Doctoral Dissertations via EBSCO host, Union Catalogue of Theses and Dissertations (UCTD) and SA ePublications via SABINET Online and World Cat Dissertations, Theses via OCLC and Google Scholar. Publications by Medical Research Council and Human Sciences Research Council were also reviewed. Websites, such as, the World Health Organization (WHO), the Joint United Nations Programme on HIV/AIDS (UNAIDS) and governmental websites and statistics institutions were also searched for policies and guidelines on HIV testing among men. We searched for published and grey literature studies from January 1990 to August 2018. We used the following search key words: HIV testing; linkage to care, Africa. Boolean terms such as ‘AND’ and ‘OR’ were also used. We included the Medical Subject Headings (MeSH) terms in the keyword search. Following keyword search, eligible studies were exported to the Endnote version 7 library for abstract screening and full article screening.

### Study selection

Abstracts and full articles screening were conducted by the two independent reviewers, MH and SM, with guidance from the eligibility criteria for this study. The eligibility criteria was designed to limit the study to focus only on the articles that address issues described in the research question: what are intervention strategies aimed at improving men’s uptake to HIV testing services in SSA? We worked closely with the University of KwaZulu-Natal library services during database searching and retrieval of articles. Studies that could not be retrieved from databases were be obtained by contacting authors. The PRISMA extension for scoping reviews (PRISMA-ScR): checklist and explanation was employed to report the screening of results.

### Eligibility criteria

#### Inclusion criteria

The following principles were used to determine articles that met this study’s inclusion criteria (1) studies presenting evidence that was published between January 1990 and August 2018; (2) studies presenting evidence on men in SSA; (3) studies that present evidence on HIV testing services among men. The above-mentioned inclusion criteria was applied to both published and grey literature. No limits were applied for language.

#### Exclusion criteria

Studies with no evidence on HIV testing services among men were excluded.

### Quality of evidence

To determine the quality of the selected studies, a Mixed Method Appraisal Tool (MMAT) version 2018 [33], was adopted and piloted by the two independent reviewers (MH and SM). The MMAT is a critical appraisal tool that is designed for the appraisal stage of systematic mixed studies reviews, like reviews that include qualitative, quantitative and mixed methods studies [33]. The tool permits to appraise the methodological quality of five categories to studies: (1) qualitative research, (2) randomized controlled trials, (3) non-randomized studies, (4) quantitative descriptive studies, and (5) mixed methods studies [33]. This tool was adopted and used in this study to appraise the above-mentioned categories, in which all included studies proved to have followed proper methodological approaches.

#### Charting the data

We developed a data collection instrument (using Google Forms) to confirm the study characteristics as well as relevance. Our data extraction tool used the following elements: (1) author(s) and date of publication, (2) aim(s) or research questions, (3) study population, (4) mean age of participants; (5) gender, (6) percentage of women, (7) percentage of men, (8) geographic setting (rural/urban), (9) study design, (10) type of Intervention and outcomes, (11) most relevant finding, (12) most significant finding, (13) study limitations and implications and (14) interpretation and conclusions from authors.

##### Collating and reporting

We used NVivo version 11 for content thematic analysing of data from the eligible articles [34].

## Results

**Fig. 1:**
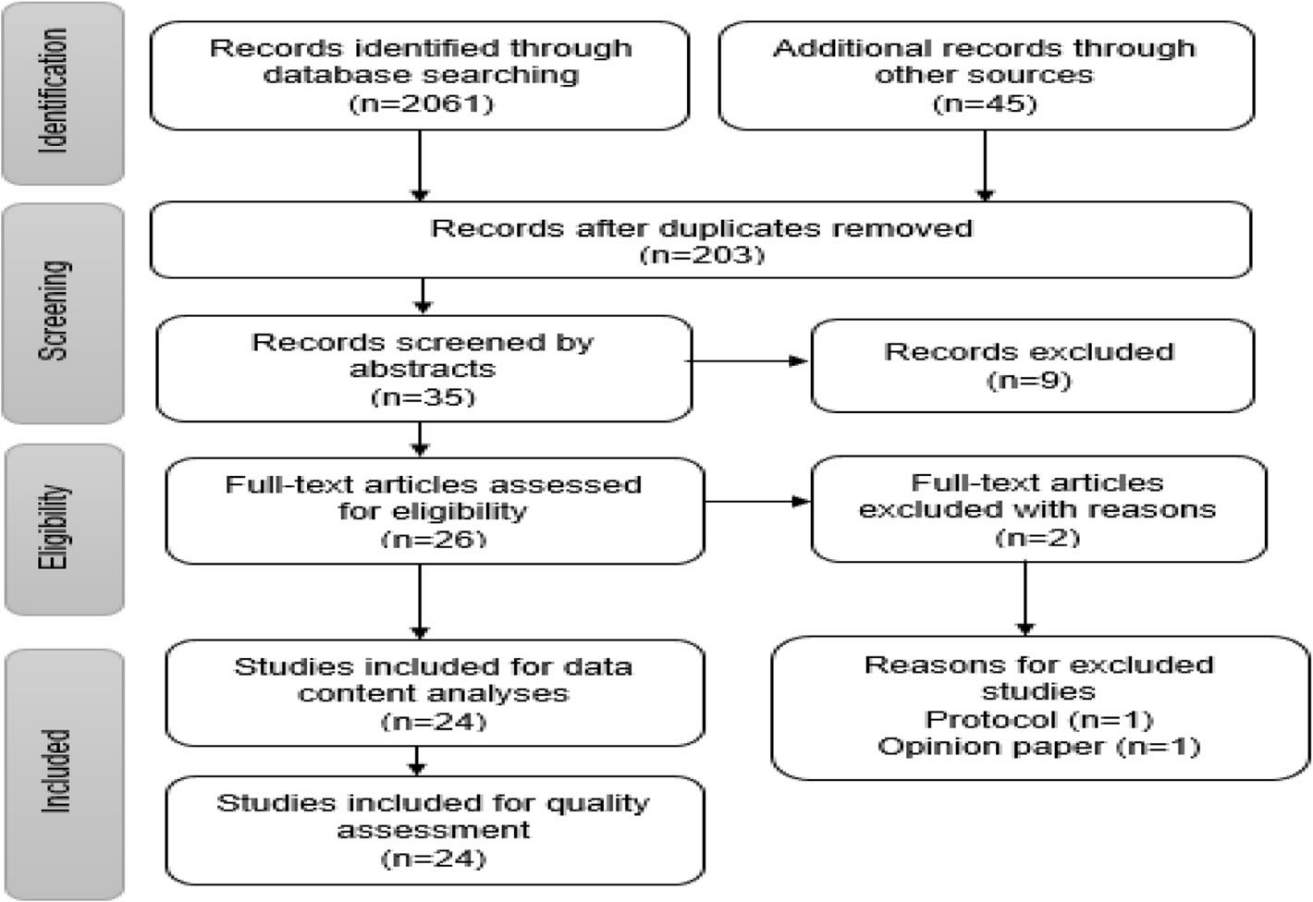
PRISMA flow chart demonstrating literature search and selection of studies

A total of 2061 articles were identified by our search criteria during the database searching stage, 45 articles were retrieved from other sources - giving us a total of 2106 articles (Fig. 1). As many as 1891 articles were not selected at database search stage because they formed part of our exclusion criteria. Twelve duplicates were removed, leaving 203 articles eligible for a further title screening stage. Of these, 35 articles were screened for abstracts, and 9 were excluded from these. Twenty six (26) articles remained for full article screening and 2 were excluded with reasons. The reasons for excluding the two studies at full article screening are as follows: one article was an opinion paper. The other article was a study protocol. Therefore, twenty four studies met our inclusion criteria and were included to quality assessment stage.

### Characteristics of included studies

**Fig. 2:**
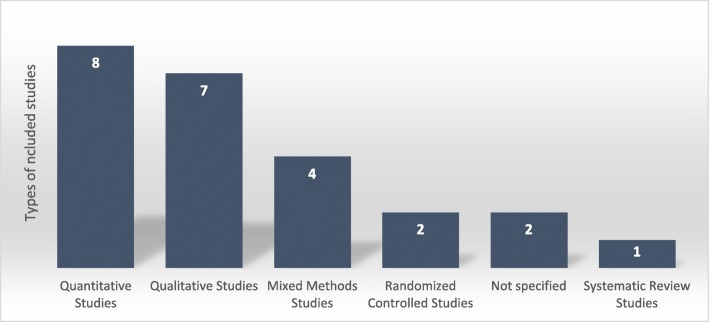
Distribution of study designs used in the included studies (*N* = 24)

**Fig. 3:**
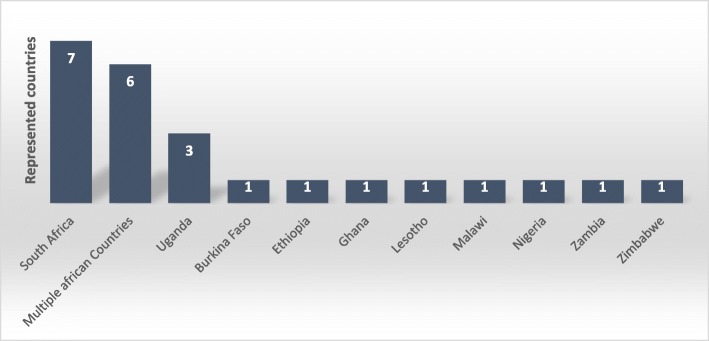
Distribution of countries represented in the included studies (N = 24)

Seven studies used qualitative research method [35–41], eight studies were quantitative [6, 7, 42–47], four were mixed methods (both quantitative and qualitative) [5, 48–50], two were randomized controlled trials [23, 51], two did not specify [22, 52], and one was a systematic review [53] (Fig. 2). More than 10 African countries were represented in the included studies (Fig. 3). The majority of included studies (83%) were published from 2010 onwards. The characteristics of included studies are also shown in Table 2.

**Table 2:** Characteristics of included studies

Author & year	Country	Study aim	Population	Sample	Age group	Study Design	Research Method
Aarnio et al., 2009 [48]	Malawi	To explore married men’s perceptions of HIV in pregnancy and male involvement in antenatal HIV testing and counselling in Southern Malawi	Men	388	15 years & older	cross-sectional survey & FGD)	quantitative & qualitative
Auld et al., 2015 [42]	Multiple (East & West Africa)	To evaluate gender equity in ART access among adults	Men & women	765,087 (patient records)	15 years & older	Patient records	Quantitative
Bwambale et al., 2008 [49]	Uganda	To determine the prevalence and factors associated with VCT use among men in Bukonzo West health sub-district, Kasese district	Men	780	18 years & older	FGD, KI interviews	quantitative & qualitative
Camlin et al., 2016 [35]	Kenya & Uganda	To explore gender, cultural factors, and community level processes that influence men’s HIV testing uptake	Men	230	Youth & adults	Observations, FGD, in-depth interviews	Qualitative
De Allegri et al., 2015 [5]	Burkina Faso	to explore factors shaping the decision to undergo Human Immunodeficiency Virus (HIV) testing among men in rural Burkina Faso	Men	937	20 & above	Survey & interviews	Quantitative & qualitative
DiCarlo et al., 2014 [36]	Lesotho	To explore gender norms, sexual decision-making, and perceptions of HIV among a sample of Basotho men and women in order to understand how these factors influence HIV testing and prevention.	Men & women	30 men; 200 women	18 years & older	interviews	Qualitative
Ezeanolue et al., 2016 [23]	Nigeria	To assess whether a congregation-based intervention, the Healthy Beginning Initiative (HBI), would lead to increased uptake of HIV testing among male partners of pregnant women during pregnancy.	Men	2498	18 years & older	Randomized controlled trial	Quantitative
Gage et al., 2005 [6]	Uganda	To examine rates and predictors of self-reported HIV testing and willingness to test among married men aged 15 /59 in Uganda	Men	1962	15–54 years	DHS	Quantitative
Harichund et al., 2018 [37]	South Africa	To assess whether men or women in KwaZulu-Nataldisplayed a higher acceptance of HIVST and also explored factors that influenced and motivatedtheir acceptability.	Men & women	12 males & 28 female	18 years & older	FGD, interviews	Qualitative
Hensen et al., 2014 [19, 53]	Sub-Saharan Africa	To describe HIV testingamong men in rural Lusaka Province, Zambia.	Men	2828	15 years & older	Systematic Review	Quantitative
Hensen et al., 2015 [51]	Zambia	To describe HIV testing among men in rural Lusaka Province, Zambia	Men	2828	15–60 years	Randomized controlled trial	Quantitative
Leblanc et al., 2015 [50]	Ghana	To explore men’s HIV knowledge, perceptions of HIV risk, and willingness to test for HIV in preparation for the initiation of formalized voluntary counselling and testing (VCT) services at Yendi Hospital in Yendi District, Ghana	Men	129	18 years & older	surveys, FGGs, interviews	quantitative & qualitative
Leichliter et al., 2011 [38]	South Africa	To explore sexual health care access and seeking behaviours in men	Men	58	18 years & older	FGD	Qualitative
Leta et al., 2012 [43]	Ethiopia	To investigate factors associated with VCT utilization among adult men since men are less likely than women to be offered and accept routine HIV testing	Men	6778	15–59 years	DHS	Quantitative
Mambanga et al., 2016 [39]	South Africa	To investigate the factors that contribute to men’s reluctance to seek HCT at primary health care facilities in Vhembe District, South Africa.	Men	15	Adult (age not specified)	semi-structured interviews	Qualitative
Matovu et al., 2014 [40]	Uganda	To explore the motivations for and barriers to couples’ HCT among married couples in Rakai, Uganda.	Men & women	157	15 years & older	FGD, KI interviews	Qualitative
Mhlongo et al., 2013 [44]	South Africa	To determine factors associated with never testing for HIV and consistent condom use among men who nevertest in Soweto.	Men	1539	18–32 years	Questionnaires	Quantitative
Nglazi et al., 2012 [7]	South Africa	To assess the effectiveness of incentivized mobile HCT in reaching unemployed men in Cape Town, South Africa	Men	9416	15 years & older	Survey	Quantitative
Remien et al., 2009 [52]	Middle East and North Africa region	To assess important gender dimensions of access to HIV testing, care and treatment in the MENA region.	Men & women	Not specified	Not specified	not specified	not specified
Sharma et al., 2017 [22]	sub-Saharan Africa	To examine community-based strategies to strengthen men’s engagement in the HIV care cascade in sub-Saharan Africa	Men & women	Not specified	15 years & older	not specified	not specified
Skovdal et al., 2011 [41]	Zimbabwe	To examine qualitatively how local constructions of masculinity in rural Zimbabwe impact on men’s use of HIV services	Men & women	78	Adult (age not specified)	FGD, interviews	Qualitative
Stephenson et al., 2013 [46]	Multiple African countries	To investigate community influences on HIV testing among men ages 15–54, using Demographic and Health Survey (DHS) data from Chad, Ghana, Malawi, Nigeria, Tanzania, Uganda, Zambia, and Zimbabwe.	Men	13,162	15–54 years	DHS	Quantitative
Huegra et al., 2018 [47]	South Africa	To assess progress towards the UNAIDS 90–90-90 targets in Mbongolwane and Eshowe, KwaZulu-Natal, South Africa.	Men & women	5649	15–59 years	Cross-sectional	Quantitative
Scott-Sheldon et al., 2013 [45]	South Africa	To evaluate the impact of testing on HIV knowledge and sexual risk among men in South Africa.	Men	819	18 years & older	Cross-sectional	Quantitative

### Quality of evidence from included studies

All of the included studies which underwent methodological quality assessment scored the high quality score between 80 and 100%. The overall evidence was considered to have minimal risk of bias.

### Study findings

The following main themes emerged from the included studies: education about HIV; Community-Based HIV testing; Home-Based HIV testing; ANC HIV testing; HIV testing incentives; HIVST; Partner HIV testing or Index testing.

### Education about HIV

Evidence on education was reported in thirteen studies, which covered more than ten countries in SSA [6, 22, 23, 36, 39, 41, 43–47, 51, 52]. Different studies indicated that such educational interventions should involve local leaders, including chiefs [36] and/or be part of the community-based outreach campaigns [22, 36, 39, 46, 52].Other studies found that having a higher HIV/AIDS-related knowledge was associated with improved engagement with HIV testing services [43] and reduced sexual risk behaviour [45]. Educational strategies for men may also work best if they are driven by other men [23, 36], in order to fight the stigma and masculinity challenges preventing men from testing for HIV [23, 43]. Educational programmes may include the basic HIV information [47], HIV transmission [6, 43], risk reduction strategies [43, 44], confidentiality [43]. For example, these may be in the form of presentations at local clinics, community gatherings or during outreach campaigns. There remain an educational gap on HIV programmes that are specifically tailored for men.

### Community-based HIV testing

Fifteen studies presented evidence on community-based HIV testing [6, 22, 23, 35, 36, 41, 42, 44, 46–51, 53]. Five studies indicated that most men preferred using community-based HIV testing services [22, 23, 36, 49, 53], as compared to facility-based testing [36, 50] due to stigma emanating from being seen at an HIV testing site [36]. Hensen et al. (2014) has demonstrated that, in SSA community-bases HIV testing had a significant effect on reaching a high number of clients who tested for HIV and the positivity rate, when compared to facility-based testing [53]. In addition to the reduced stigma associated with community-based HIV testing services, this strategy also reduces the financial costs associated with travelling to facilities and improves access to HIV testing services among men of lower socio-economic status [51]. It also helps reach younger men as well [47, 48]. Although community-based HIV testing reduces the stigma associated with HIV testing, it does not eliminate barriers to men’s uptake to HIV testing services completely [35]. Clients still have to travel to clinics and wait in long queues to obtain ART after testing HIV positive [22]. Sharma et al. (2017) has demonstrated that the incorporation of the ‘test and treat’ strategy as part of the community-based HIV testing services may help reduce loss to follow-up associated with facility-based ART initiations [22]. A study conducted in Uganda, concluded that expanding community mobile clinics throughout the country and rural communities, is essential to attract men to HIV testing services [6] as well as earlier linkage to ART services [22]. Sharma et al. (2017) and Stephenson et al. (2013) recommend the need for tailored community-based HIV services to support the needs of men, including flexible hours, convenient and private access to care and multiple follow-up visits [22, 46]. Despite the robust evidence on the benefits to community HIV testing among men in SSA, there remains a gap as far as ART initiations of HIV positive clients found through this community-based testing.

### Home-based HIV testing

Evidence on home-based testing as a strategy for improving men’s HIV uptake was reported in seven articles, representing more than ten SSA countries [22, 23, 36, 38, 40, 51, 53]. Studies conducted in Lesotho, Nigeria, Zambia and South Africa revealed that men are more open to HIV testing when it is conducted at home [23, 36, 38, 51], due to fear of being stigmatised in clinic settings [36], as well as judgements by healthcare workers [38]. The supervised home-based counselling and testing services has been shown to be especially effective [36, 51]. Two studies further indicated that home-based testing will not only improve men’s uptake to HIV testing, but the strategy will further help promote couple’s testing [40, 53], as well as HIV testing outcomes discloser among couples [36]. As high as 53% (*n* = 1499) of men accepted home-based HIV testing services in a study conducted in rural Zambia [51]. Although home-based testing has been found to attract more men for HIV testing services, another study revealed that home-based HIV testing reaches lesser numbers of men when compared to mobile testing [53]. This study further indicated that despite the improved number of men’s HIV uptake during home-based testing, the impact at which this strategy is making, become limited when it is conducted among highly mobile communities, because men are less likely to be at home due to employment [53]. While home-based HIV testing strategy is effective, there remain challenges for HIV uptake especially posed by mobile populations.

### ANC HIV testing

Evidence on ANC HIV testing as a strategy for improving men’s HIV uptake was reported in nine articles, covering more than eight SSA countries [5, 22, 23, 38, 40, 48, 49, 51, 53]. This strategy is sometimes referred to as ‘Partner testing’ [38, 51]. At least four studies revealed that ANC acts as an important tool for improving men’s HIV uptake [22, 38, 49, 53], although only a few of them benefit from this strategy [5, 23, 48]. As high as 72% of study participants of a study conducted in Malawi were open to receiving HIV testing information and advices in the company of their wives, while only 14 and 13% preferred receiving the same information in the presence of peers or alone, respectively [48]. In support of this, another Ugandan study indicated that as high as 91% of interviewed study participants supported HIV testing with their wives or partners [49]. While this strategy has the ability to improve men’s HIV uptake, a study by Aarnio, further indicated that this strategy’s shortcoming is that very few men accompany their wives or partners to clinics for antenatal visits [48], with the rates ranging between 1.8 to 32% in the SSA region [23]. Only 25% of men in a study conducted in Burkina Faso tested for HIV during ANC visits as a result of their wives’ request [5]. Challenges of mistrust between couples, lack of HIV awareness and fear were cited as the main factors affecting men’s participation in their wives’ or partners’ HIV testing during ANC visits [23]. Despite the ANC HIV testing potential to increasing men’s HIV uptake, there remain a coverage gap given the lower numbers of men this strategy can attract.

### HIV testing incentives

Five studies reported evidence on providing incentives to improve HIV uptake among men [7, 35, 36, 41, 51]. These studies were conducted in six different SSA countries. These incentives may be in a form of money, food, calling cards or t-shirts [36]. This HIV testing strategy is supported by Hensen et al. (2015) study, which indicated that these incentives may either be in the form of finance or material, given the impact these may have, especially if used as in the ANC to link men into HIV testing after their female partners have been tested in clinics [51]. While Hensen et al. (2015) study recommends incentives as an effective strategy during ANC visits, another study conducted in South Africa found that an incentive HIV testing strategy does not only improve men’s HIV uptake but also HIV testing positivity yield in mobile settings, especially when these are compared with clinic-based HIV testing services [7]. As high as 15% positivity rates were found through incentives in mobile clinic as compared to the usual 8% when incentives were not involved [7]. Again, 60% of first-time men testers were reached using incentives versus the usual 42% [7]. Incentives do increase men’s HIV uptake even at clinics settings specifically the Community Health Centres (CHCs) [35]. Although incentives have been noted to improve men’s HIV uptake and positivity rates, there remains ethical concerns towards this strategy, as it exposes people to bribery, autonomy and client coercion [7, 41]. Incentivised HIV testing strategies remain controversial in SSA.

### HIV self-testing

Evidence on self-testing as a strategy to improve HIV testing among men was reported in four articles [22, 37, 51, 53]. Some of these studies revealed that HIVST is capable of improving HIV uptake among men [22, 37, 53], because it is cost-effective [22, 37], confidential and convenient [37]. This HIV testing strategy was noted as effective and an alternative strategy to facility based testing due to long queues and waiting times experienced in these settings [37]. This study further revealed that HIV-self testing helps address men’s discomforts, which occur as a result of engaging with healthcare workers [37]. While this strategy removes many HIV testing barriers among men, it also improves men’s autonomy [51], which addresses masculinity issues. HIV self-testing strategy has as high as 70 to 94% of acceptability rates [37]. Although HIV-self testing is suggested as a viable alternative for improving HIV uptake among men in Africa, however, this HIV testing strategy, has its own limitations. These limitations include the fact that confirmatory tests are still required after using the HIV-self testing kits [22]. Although this is the case, HIV-self testing strategy remain one of the prominent strategies to improve HIV testing uptake in Africa, even among high risk men [22, 37]. More research is needed to establish and improve the accuracy of self-test kits (given the confirmatory test required after using HIVST); monitoring of test kits and LTC of clients after testing HIV positive.

### Partner HIV testing or index testing

Partner HIV testing as a strategy to encourage men on HIV uptake was discussed in four articles, covering more than five SSA countries [22, 23, 39, 40]. Partner testing, otherwise known as index testing is the process of notifying partners of newly diagnosed HIV-positive clients [22, 23]. According to a study conducted in Uganda, partner testing is likely to reduce mistrust and increasing faithfulness among partners [40]. In SSA, the partner testing strategy reached more men for HIV testing services when using active notification (contract referral or provider tracing) (50%) versus the passive notification in which index cases are asked to refer their sexual partners to the clinics for HIV testing services (15%) [22].

## Discussion

This study aimed at mapping evidence on the strategies and interventions to improving men’s HIV uptake among men in SSA. This study revealed education about HIV; Community-Based HIV testing; Home-Based HIV testing; ANC HIV testing; HIV testing incentives; HIVST and Partner HIV testing or Index testing as important interventions to improving HIV testing among men in SSA. Implementing strategies or interventions to improve HIV uptake on men has also been stressed by the UNAIDS as one of the critical strategic goals [54]. Addressing the first 90 of the 90–90-90 HIV cascade has been raised by the UNAIDS as an important priority, with educating men about HIV being at the forefront [54].

Strategies and interventions to improving HIV testing among men were demonstrated in the following countries: South Africa, Uganda, Zimbabwe, Nigeria, Zambia, Ethiopia, Lesotho, Kenya, Burkina Faso and a combination of Chad, Ghana, Malawi, Tanzania, Uganda, Zambia and Zimbabwe, as well as SSA region. Our study findings show that home-based HIV testing strategy to attract men, is important and useful, however, the chances of accessing a bigger number of men is limited, especially when compared to community-based HIV testing [53]. While community based HIV testing has been seen to help access more men, this study revealed that the implementation of the ‘universal test and treat’ strategy to this strategy may reduce loss to follow-up associated with facility-based ART initiations [22]. Our study further revealed that ANC HIV testing has a potential to increasing men’s involvement to HIV testing during their wives or partners’ ANC visits, however, fewer men present themselves with their partners in clinics [5, 22, 23, 40, 48]. Despite the ethical issues, our study found that providing incentives improves not only HIV testing among men but also the HIV positivity rates [7, 35, 41]. While education is key to changing men’s perspectives and improving HIV knowledge, this study further revealed that the HIVST strategy is critical to addressing issues of confidentiality and convenience [22, 37], as well as autonomy among men [51]. Therefore, this strategy is key to responding to the UNAIDS plans to addressing masculinity issues as far as men’s engagement to HIV testing is concerned [54].

The findings of this study are consistent with the findings of other studies conducted in resource limited settings, where interventions aimed at improving HIV testing, such as, ANC HIV testing [55, 56], community [57–60] and home-based HIV testing [57, 61–64], HIVST [65–68], as well as, incentivised approaches [69], showed positive outcomes. Given the many barriers associated with HIV testing in a clinic setting, more men currently prefer to engage themselves with testing services that are conducted outside this setting [70–74]. Despite our findings supporting the implementation and strengthening of these strategies to attract more men to HIV testing in SSA it should be noted that our findings also revealed some of the pitfalls in some of these strategies. Therefore, while these are implemented and/or expanded, it is important for implementers to work towards improving linkage to ART initiation after home-based or community-based testing services [22], as well as, the ethical issues pertaining to incentivised HIV testing services [7, 41] and the quality of HIV self-tests.

### Strengths and weaknesses

All included studies underwent quality appraisal using an approved tool, the MMAT. Our full article screening tool was piloted to ensure the reliability of included studies as demonstrated by the degree of agreement results. Analysis of the results of full article screening show that there was 95.24% agreement versus 95.24% expected by chance which constitutes a considerably high agreement between screeners (Kappa statistic = 0. 00 and *p*-value < 0.05) (Additional file 1). In addition, the McNemar’s chi-square statistic suggests that there is not a statistically significant difference in the proportions of yes/no answers by reviewer with p-value > 0.05. Although our title screening included a wide range of databases, the overall search strategy may have been biased toward public health and social sciences. Searching other bibliographic databases may have yielded additional published studies. Despite the generally relevant key words/terms used while searching for relevant articles in different databases, other terms may also exist as reference to HIV testing. While our review included any article published in any language, our search was conducted using only English terms. Despite these limitations, we believe that our search strategy was comprehensive in reviewing the public health and social sciences literature on the strategies and interventions to improving HIV testing among men in SSA.

### Recommendations for future research

Our study findings show that the HIVST strategy is one of the preferred interventions by many men [75] and it has been shown to get more men testing for HIV in similar settings [76–79]. However, more research is needed to establish the accuracy of test kits (given the confirmatory test required after using HIVST); monitoring of test kits and LTC of clients after testing HIV positive. Our study findings further show that there is limited published systematic scoping reviews specific to streamlining the effective HIV testing strategies, in order to address the first 90 of the 90–90-90 HIV cascade, particularly among men in SSA.

### Implications for practice

We concur that strategies aimed at improving men’s HIV testing at the comfort of their homes and through community or mobile clinics should be strengthened and expanded to attract more men [7, 46]. However, we further recommend that community mobile clinics should also initiate clients tested HIV positive on ART services, otherwise direct LTC challenges will remain. We further believe that attracting men at taxi ranks and close to their work places may also be beneficial towards addressing the first 90 of the HIV cascade, especially because men in general prefer testing outside of the clinic setting [44].

## Conclusion

The findings of the study indicate that Community-Based HIV testing; Home-Based HIV testing; ANC HIV testing; HIV testing incentives; HIVST and Partner HIV testing are feasible intervention strategies to improving men’s HIV uptake in SSA. We conclude that these interventions and strategies should be expanded and strengthened to ensure more men are diagnosed for HIV and initiated on ART services without delays. While these programmes are implemented, it is important that barriers associated with incentives, HIVST and the LTC gap, as a result of home and community-based HIV testing, are reduced.

## Additional file

Additional file 1: Full article screening results (DOCX 13 kb)

## Data Availability

All the data analysed and reported in this paper was from published literature, which are already in the public domain. The raw data can be accessed through Table 2, which characterises the included studies.

## Abbreviations

AIDS: Acquired Immunodeficiency Syndrome
ANC: Antenatal Care
ART: Antiretroviral treatment
HIV: Human Immunodeficiency Virus
HIVST: HIV self-testing
LTC: Linkage to care
MeSH: Medical Subject Headings
PCC: Population, Concept and Context
PITC: Provider initiated testing and counselling
SSA: Sub-Saharan Africa
UCTD: Union Catalogue of Theses and Dissertations
UNAIDS: Joint United Nations Programme on HIV/AIDS
WHO: World Health Organization
