# Supplementary material for: Mapping evidence of intervention strategies to improving men’s uptake to HIV testing services in sub-Saharan Africa: A systematic scoping review
Source: BMC Infect Dis. 2019 Jun 6;19:496. doi: 10.1186/s12879-019-4124-y (PMC6554953; doi:10.1186/s12879-019-4124-y)
Supplement: Supplementary file 1 — Full article screening results (DOCX 13 kb) [file 12879_2019_4124_MOESM1_ESM.docx]

**Additional file 1:** Full article screening results

| **No.** | **Author and Date** | **Screener_1** | **Screener_2** |
| --- | --- | --- | --- |
| 1 | Aarnio et al, 2009 | 1 | 1 |
| 2 | Auld et al, 2015 | 1 | 1 |
| 3 | Bwambale et al, 2008 | 1 | 1 |
| 4 | Camlin et al, 2016 | 1 | 1 |
| 5 | De Allegri et al, 2015 | 1 | 1 |
| 6 | DiCarlo et al, 2014 | 1 | 1 |
| 7 | Ezeanolue et al, 2016 | 1 | 1 |
| 8 | Gage et al, 2005 | 1 | 1 |
| 9 | Harichund et al, 2018 | 1 | 1 |
| 10 | Hensen et al, 2014 | 1 | 1 |
| 11 | Hensen et al, 2015 | 1 | 1 |
| 12 | Huegra, 2018 | 1 | 1 |
| 13 | Leblanc et al, 2015 | 1 | 1 |
| 14 | Leichliter et al, 2011 | 1 | 1 |
| 15 | Leta et al, 2012 | 1 | 0 |
| 16 | Mambanga et al, 2016 | 1 | 1 |
| 17 | Matovu et al, 2014 | 1 | 1 |
| 18 | Mhlongo et al, 2013 | 1 | 1 |
| 19 | Nglazi et al, 2012 | 1 | 1 |
| 20 | Remien et al, 2009 | 0 | 1 |
| 21 | Sharma et al, 2017 | 1 | 1 |
| 22 | Scott-sheldon, 2013 | 1 | 1 |
| 23 | Skovdal et al, 2011 | 1 | 1 |
| 24 | Stephenson et al, 2013 | 1 | 1 |

**Stata output (Put this in your appendices)**

. kap Screener_1 Screener_2

Expected

Agreement Agreement Kappa Std. Err. Z Prob>Z

-----------------------------------------------------------------

95.24% 95.24% 0.0000 0.0000 . .

. mcc Screener_1 Screener_2

| Controls |

Cases | Exposed Unexposed | Total

-----------------+------------------------+------------

Exposed | 20 1 | 21

Unexposed | 0 0 | 0

-----------------+------------------------+------------

Total | 20 1 | 21

McNemar's chi2(1) = 1.00 Prob > chi2 = 0.3173

Exact McNemar significance probability = 1.0000

Proportion with factor

Cases 1

Controls .952381 [95% Conf. Interval]

--------- --------------------

difference .047619 -.0910823 .1863204

ratio 1.05 .9542341 1.155377

rel. diff. 1 1 1

odds ratio . .025641 . (exact)
